# Ovarian follicle dynamics in the plains zebra (*Equus quagga*)

**DOI:** 10.1371/journal.pone.0348772

**Published:** 2026-05-22

**Authors:** Lawrence O. Barros, Kendall A. Hyde, Nathalie Gazzaniga, Melba O. Gastal, Francisco L. N. Aguiar, Gustavo F. Carneiro, Eduardo L. Gastal

**Affiliations:** 1 Equine Reproduction Veterinarian, João Pessoa, Paraíba, Brazil; 2 Animal Science, School of Agricultural Sciences, Southern Illinois University, Carbondale, Illinois, United States of America; 3 Department of Veterinary Medicine, Sousa Campus, Federal Institute of Education, Science and Technology of Paraíba, Sousa, Paraíba, Brazil; 4 Department of Veterinary Medicine, Federal Rural University of Pernambuco, Recife, Pernambuco, Brazil; UPR: University of the Poonch Rawalakot, PAKISTAN

## Abstract

Understanding follicular dynamics during the estrous cycle is essential for developing breeding programs and assisted reproductive technologies (ARTs) in any species. In this context, knowledge of follicular dynamics and related uterine changes in the plains zebra (*Equus quagga*) is essential to increase the declining population of this vital African species and to provide comparative insights into more endangered zebra species. Likely due to the difficulty of handling these wild species, no detailed reproductive study using ultrasonography has been performed in any zebra. Therefore, the present study aimed to describe follicular dynamics and endometrial echotexture changes in plains zebras using data collected daily via transrectal ultrasonographic monitoring of the ovaries and uterus during spontaneous estrous cycles (no exogenous hormones administered; Experiment 1) and induced ovulatory periods (exogenous hormones administered; Experiment 2). Data from four plains zebra mares revealed that their spontaneous estrous cycles (n = 9) are characterized by a long interovulatory interval (IOI; approximately 38 days), low daily antral follicle count during the IOI (approximately 3 follicles), an absence of follicular waves, a slow follicular growth rate (approximately 1 mm per day), and a relatively similar mean ovulatory follicle diameter (approximately 36 mm) compared to domestic equids. Furthermore, the zebra shows endometrial echotexture patterns similar to those of the horse mare during the preovulatory period. Additionally, by evaluating induced ovulatory periods in two zebras (n = 10 periods), it was shown that these animals respond reliably to luteolytic and ovulation synchronization drugs. Overall, the information provided in this study is vital for the development of breeding programs and ARTs in the plains zebra. In this way, conservation efforts can not only begin to improve the numbers of the plains zebra but also use the information learned from this species to inform efforts for other, more endangered zebras.

## Introduction

Although the zebra plays a significant cultural and ecological role as a keystone species in the eastern and southern African savannas, studies of its reproductive physiology are scarce. Furthermore, most fundamental knowledge of zebra reproductive physiology has been derived from post-mortem examinations [1,2], observational data on parturition and breeding behavior [3,4], and fecal and urinary hormone analyses [5]. To date, there are three species of extant zebra: the plains zebra (*Equus quagga*), mountain zebra (*E. zebra*), and Grévy’s zebra (*E. grevyi*); however, all zebra species face challenges critical to their survival [6–8]. As a grazing herbivore, the plains zebra promotes biodiversity through maintenance and growth regulation of savannas and grasslands and by serving as a vital food source for large predators [9]. While the plains zebra is not currently classified as endangered, this species still faces critical challenges to its survival, as many zebras are hunted or poached for their valuable, distinctive hides, while others are greatly affected by habitat fragmentation due to human intervention and competition with livestock animals [6,9]. In this way, conservation efforts, including breeding management programs in and out of captivity, are essential to ensure the survival of this vital African species.

Because studies on the reproductive physiology of zebra mares are limited, most information regarding ovarian, follicular, and uterine dynamics during the estrous cycle is assumed from other, better-studied domestic equids such as the horse and donkey. However, even within these domestic species, variation exists between the horse mare and jenny [10,11] and between breeds of horse mare [12]. Nonetheless, reproductive cyclicity in both horse mares and jennies is characterized by waves of synchronic emergence, growth, and regression of several ovarian follicles (structures that house the egg cell or oocyte), sometimes accompanied by selection of a dominant follicle that may ovulate [10,11,13]. Several types of these follicular waves have been characterized in horse mares and jennies, including major waves, where a follicle is selected and achieves dominance while subordinate follicles regress, and minor waves, where dominance does not occur [10,11,14,15]. Major waves can be further subdivided into primary waves, where the dominant follicle ovulates, or secondary waves, where the dominant follicle regresses [14]. In horse mares and jennies, there can be 1–3 follicular waves per interovulatory interval (IOI; the time interval between two consecutive ovulations), with each IOI lasting 21–26 days on average, depending on many factors, including, but not limited to, species, breed, and time of year [10–12,15,16]. For these domesticated equid species, emergence is generally defined as the day when a group of follicles reaches 6 mm in diameter under increasing concentrations of follicle stimulating hormone (FSH; [10,13]). In a minor wave, these follicles will then regress after some time of synchronized growth before the dominant follicle is selected; however, in a major wave, the largest follicles will grow simultaneously during the common growth phase until deviation, where selection occurs and the dominant follicle continues to grow while the remaining subordinate follicles grow at a reduced rate and regress [10,14]. In a primary wave, follicular deviation occurs in conjunction with a decreasing systemic concentration of FSH and increasing concentration of systemic luteinizing hormone (LH; [13,17]). As a consequence, LH systemic concentration continues to increase after deviation to facilitate the establishment of a dominant follicle [18] and peaks at one day post-ovulation [19,20]. In both horse mares and jennies, the dominant follicle has a growth rate of around 2–3 mm per day after selection, reaching a maximum size of 35–45 mm before ovulation, again depending upon species, breed, age, and other factors [11,12,15,16,21].

Understanding these key follicular development time points in the equid estrous cycle are vital for the design and proper enactment of breeding protocols for assisted reproductive technologies (ARTs), such as artificial insemination (AI), embryo transfer, ovum pick-up (OPU), and cryopreservation of gametes and embryos [22–24]. However, the development of ARTs in zebra species has been slow compared to that in domestic equids, likely due to the difficulty of safe and effective restraint and the lack of knowledge of their reproductive physiology [25]. Despite these challenges, several ART techniques have been studied in the zebra, including OPU and *in vitro* fertilization (IVF; [26]), somatic cell nuclear transfer (SCNT; [27]), and embryo transfer [28]. In fact, one live plains zebra foal was born as a result of embryo transfer to a Welsh pony mother [28]. Nonetheless, even more basic and less invasive ARTs, such as AI protocols, have yet to be fully developed for the zebra due to limited information on reproductive physiology. As such, the plains zebra is a particularly appealing zebra species to study, as the higher population of plains zebras (150,000–250,000 mature individuals; [6]) can be used as a model species to study the more endangered Grévy’s (<2,000 mature individuals; [7]) and vulnerable mountain (<35,000 mature individuals; [8]) zebras. Therefore, studies evaluating the estrous cycle of the plains zebra mare and the effects of exogenous drugs commonly used in ARTs (e.g., prostaglandins and human chorionic gonadotropin) are essential for developing safe, effective, and practical assisted reproduction methods in this critical species.

Using the available information concerning the follicular dynamics of the horse mare and the jenny, the present study hypothesized that (i) while comparable to the horse mare and jenny, the plains zebra mare displays distinct differences regarding the estrous cycle and follicle dynamics, and (ii) the plains zebra mare can respond appropriately and reliably to luteolytic and ovulation induction drugs. In this regard, the present study successfully describes and compares, for the first time, the follicular dynamics and endometrial echotexture changes of plains zebras (*E. quagga*) using data collected via transrectal ultrasonographic monitoring of ovaries and uterus under spontaneous estrous (no exogenous hormones administered; Experiment 1) and induced ovulation (exogenous hormones administered; Experiment 2) cycles. In this way, key stages of the zebra estrous cycle (i.e., emergence, maximum diameter, ovulation, etc.), follicular dynamics over time, and the effectiveness of a putative short-cycling and ovulation induction protocol in live animals have been evaluated.

## Materials and methods

### Animals

**Fig 1:**
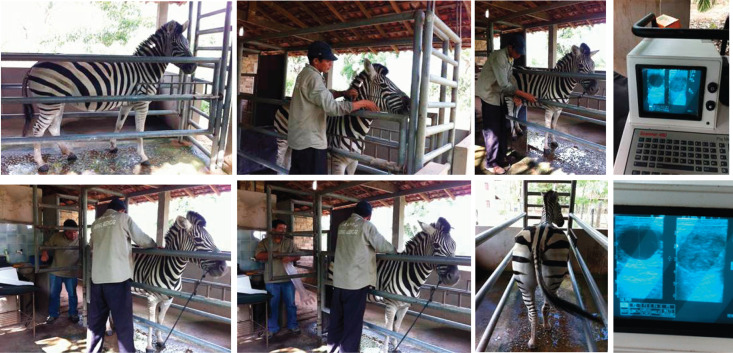
Representative images of the transrectal ultrasonography procedure in plains zebra (*Equus quagga*) mares. After 16 months of intensive training, the zebra mares (pictured: “Pepita”) were able to be loaded into the palpation stock, haltered, tied, palpated, and scanned ultrasonographically. For each scanning procedure, a handler stayed by the zebra’s head, while the ultrasound operator would transrectally palpate and then scan the animal. Images of the transrectal ultrasonography scanning are shown, with a preovulatory follicle seen on the left of the ultrasound monitor and a cross-section of the uterus shown on the right. Ultrasonography procedures of all zebra mares occurred daily without sedation and lasted approximately 5–10 min per zebra. The ultrasonographer pictured in this figure has given written informed consent (as outlined in *PLOS ONE*’s consent form) to publish their likeness in this manuscript. Pictures printed with permission from Lawrence O. Barros, original copyright 2011.

This study was conducted at a private zoo (IBAMA/CTF: 6476876; IBAMA/P-356) in Brazil (03º 40’ 00“ S, 45º 40’ 00” W) over a 24-month period. Healthy, cyclic plains zebra mares (*Equus quagga*; n = 4), aged between 5 and 8 years (mean, 6.5 ± 0.6 years), with weights averaging 375.0 ± 15.5 kg (range, 350–420 kg), and in good body condition (scores 6–7; [29,30]) were utilized. All four zebras had previously given birth to 1–2 live foals at least one year prior to the start of data collection and were not lactating at the time of the experiments. Each zebra received approximately 12 months of training to adapt to human handling and enter the palpation stock. Subsequently, the zebras underwent an additional 4 months of training in the palpation stock to allow for safe transrectal palpation and ultrasonographic examinations. Pictures of the ultrasonography process for the zebras are shown (Fig 1). Data collection via ultrasonography commenced only after these 16 months of intensive training and was performed without sedation for any zebra. All data were collected between April and November using transrectal ultrasonography, with no notable seasonal effect observed. Throughout the experiment, all animals were kept together under the same management conditions on natural pasture with *ad libitum* access to water and mineral salt, and were supplemented with 3 kg of balanced grain per day, provided in 1-kg increments throughout the day. Furthermore, no animals were exposed to lighting to induce an artificial photoperiod. All research in this study was approved by the Institutional Research Ethics Committee (CEUA IFPB #: 23000.003528.2025−01) at the Department of Veterinary Medicine, Sousa Campus, Federal Institute of Education, Science and Technology of Paraíba, Brazil.

### Experimental design and ultrasound scanning

All zebras were examined transrectally daily using a B-mode ultrasound (Pie Medical 480; PieMedical Imaging, Maastricht, Limburg, Netherlands) equipped with a linear-array transducer (5 MHz) to evaluate the reproductive tract. No sedative, spasmolytic agent, or caudal epidural anesthesia was used during the examinations. Transrectal palpation and ultrasound exams were performed by the same experienced operator and lasted approximately 5–10 min per zebra. In this study, two experiments were conducted. Experiment 1 aimed to characterize follicular dynamics during spontaneous estrous cycles in 4 zebras (“Devassa,” “Maravilha,” “Pepita,” and “Vitoria”) across two to three consecutive interovulatory intervals (IOIs). Experiment 2 aimed to assess the feasibility of short-cycling estrous cycles and ovulation synchronization, as well as to evaluate follicular dynamics and ovulation time after exogenous hormone injections in 2 zebras (“Pepita” and “Vitoria”) over five non-consecutive ovulation induction cycles. Animals were evaluated in the same order daily throughout the experimental period to prevent time-lag effects in data collection.

### Short-cycling estrous cycles and ovulation synchronization

In Experiment 2, 5 ovulation induction cycles for 2 zebras (n = 10 cycles total) were evaluated after 1.0 ml of a synthetic analogue of prostaglandin (PG; dinoprost tromethamine; Lutalyse, 5 mg/ml im; Zoetis, Parsippany, NJ, USA) was administered intramuscularly on a random day when a follicle of at least 19 mm was present. Once the largest follicle reached between 25 and 37 mm in the presence of moderate uterine edema (endometrial echotexture score = 2 out of 4; [31]), 1,500 IU of human chorionic gonadotropin (hCG; Vetecor; 1,000 IU/ml iv; Hertape-Calier, São Paulo, Brazil; [32]) was given intravenously.

### Evaluation of ovarian dynamics

#### Follicle tracking and measurement.

During each ultrasound exam, structures such as antral follicles, corpus luteum (CL), and corpus albicans observed in each ovary were recorded in a proper notebook. The retrospective location of follicles, CL, and corpus albicans was used as a reference to identify and monitor the development of the antral follicles [33]. To expedite each ultrasound exam, CL and corpus albicans were not tracked or measured daily. Daily follicle tracking began when a follicle reached a diameter of ≥2 mm. This step was essential for properly characterizing early follicle emergence and follicular dynamics. Diameters of follicles and CL (only on the day of ovulation) were measured using the average height and width of the maximum visible area of the antrum and luteal tissue, respectively [33].

#### Follicle emergence, deviation, and growth rates.

Emergence of the future dominant/ovulatory follicle was defined as the day when the follicle reached 6 mm in diameter with continued growth afterward; meanwhile, follicle deviation was identified as the day when the two largest follicles of an expected ovulatory wave showed different growth rates [13,18]. The growth rate of the future dominant/ovulatory follicle was calculated by dividing the total of daily diameter changes by the number of days measured. Ovulation was marked by the disappearance of the preovulatory follicle and the appearance of a recent CL in ultrasound images during subsequent examinations [33].

#### Identification of follicular waves and follicle classification.

After inspecting the data, the presence of any follicular wave was examined, focusing on the total number, frequency, and types of follicular waves during each IOI [33]. For follicles to be considered part of the same wave, they must emerge together within a 2–3 day period after the emergence of the future dominant follicle [17,18], and they must continue to grow alongside the future dominant follicle for at least 5–7 days after their emergence.

Because only a small number of follicles are detected daily in zebras throughout the IOI, the three largest follicles (F1, the largest; F2, the second largest; and F3, the third largest) per animal were plotted for each day. Additionally, all visible follicles measuring ≥2 mm in diameter were counted and grouped into 6 size classes: 2–5 mm, 5.1–10 mm, 10.1–15 mm, 15.1–20 mm, 20.1–25 mm, and ≥25 mm.

### Evaluation of uterine edema

The ultrasound endometrial echotexture assessments were performed daily by the same operator, blind to the stage of the estrous cycle of each zebra. The assessment of uterine edema was performed by scoring the endometrial echotexture (range, score 1 = minimum to score 4 = maximum; [34]) through ultrasonographic visualization of the longitudinal section of the uterine body and cross-sectional images of both uterine horns.

### Statistical analyses

All statistical analyses were performed using SAS statistical software (version 9.4-TS1M8, 2023, SAS Institute, Cary, NC, USA). The Kolmogorov-Smirnov test was used to verify the normal distribution of the data set. For end points that were not normally distributed, the data were transformed using rank or log_10_. All normal data or data that were normal after transformation were analyzed using one-way ANOVA, followed by *post hoc* Tukey’s tests when applicable. Any data found to be non-normal after transformation were analyzed using nonparametric tests (Kruskal-Wallis and *post hoc* Dunn’s tests). Data were expressed as mean ± SEM, unless otherwise indicated. A probability of *P* < 0.05 (two-sided) indicated that a difference was significant, and *P* > 0.05 and < 0.1 indicated that a difference approached significance.

## Results

### Ultrasonographic examination

**Fig 2:**
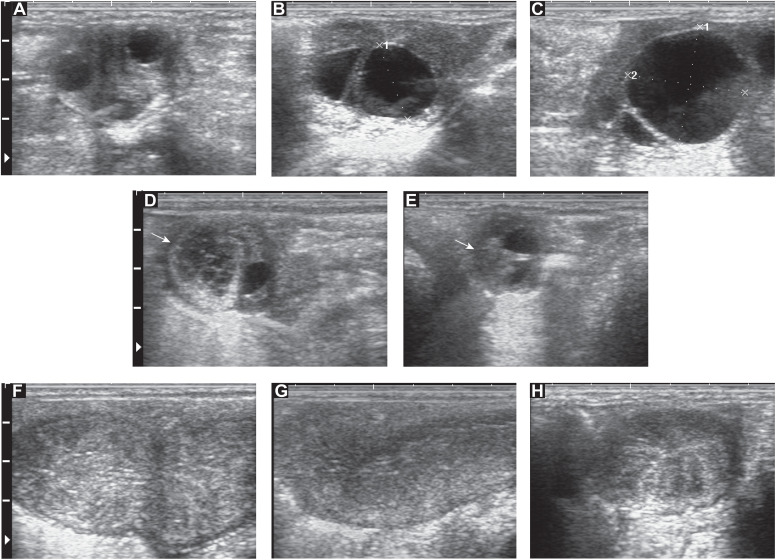
Representative images of ultrasonographic structures in the plains zebra (*Equus quagga*) mare reproductive tract. Antral follicles at different stages can be seen, including (A) early growing follicles, (B) a future dominant follicle next to a small follicle, and (C) a preovulatory follicle. (D) A large, early corpus luteum (CL; arrow) can be seen next to a small follicle. (E) A regressing CL (arrow) is pictured. The uterus is shown in different stages of the zebra estrous cycle with the (F) bifurcation of the uterine horns during diestrus, (G) uterine body during diestrus, and (H) a cross-section of the uterine horn during estrus.

Ultrasonographic images of zebra follicles (Fig 2A–2C), corpora lutea (Fig 2D–2E), and uterus (Fig 2F–2H) are shown. Follicles at different stages can be seen, including early growing follicles (Fig 2A), a future dominant follicle next to a small follicle (Fig 2B), and a preovulatory follicle (Fig 2C). A large, early corpus luteum (CL) can be seen next to a small follicle (Fig 2D). A regressing CL (arrow) is pictured (Fig 2E). The uterus is shown in different stages of the zebra estrous cycle with the bifurcation of the uterine horns during diestrus (Fig 2F), the uterine body during diestrus (Fig 2G), and a cross-section of the uterine horn during estrus (Fig 2H).

### Experiment 1. Spontaneous estrous cycles

#### Follicle dynamics.

**Fig 3:**
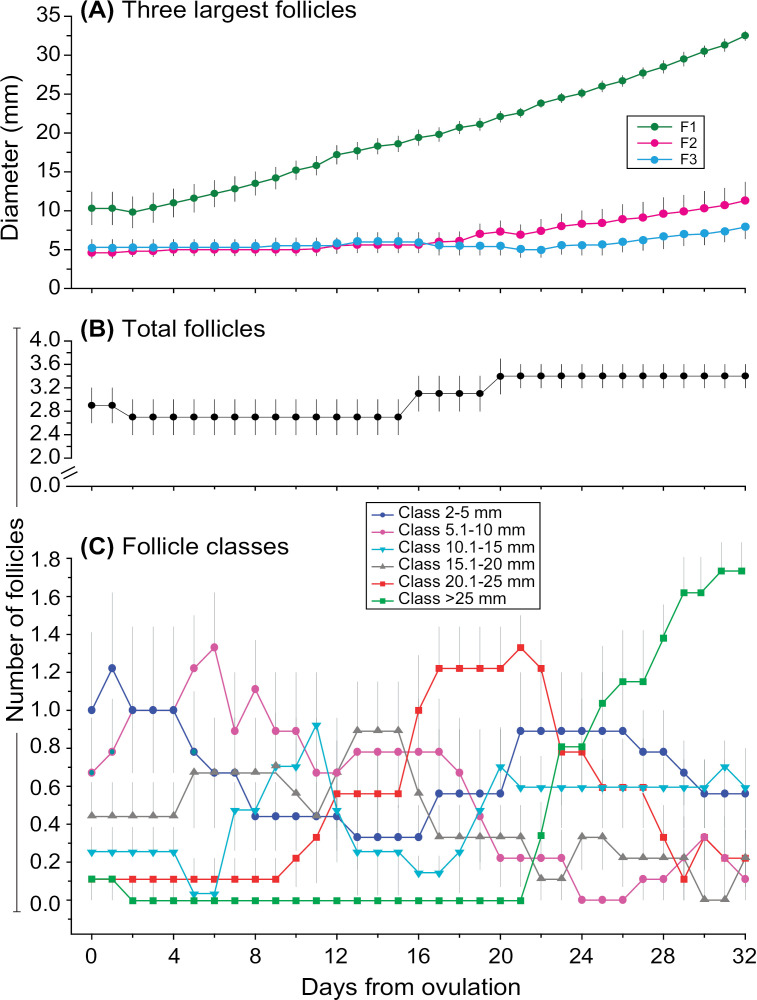
Diameter of the three largest follicles and numbers of total follicles and follicles of different diameter classes during the interovulatory interval of plains zebra mares (*Equus quagga*). Mean (± SEM) (A) diameters of the three largest follicles and (B, C) numbers of (B) total follicles and (C) follicles of different diameter classes observed during spontaneous estrous cycles in plains zebra mares (n = 9 cycles in 4 zebras) are shown. (A) Follicles were ranked by size without day-to-day identity. (A, C) Different colors indicate different-sized follicles. The length of the interovulatory interval for all cycles was truncated at Day 32 for statistical comparison. F1, first largest follicle; F2, second largest follicle; F3, third largest follicle.

The mean intervals (in days) of IOIs (ovulation to ovulation) and key stages of follicular development (ovulation to emergence, emergence to maximum diameter, emergence to ovulation, etc.), diameters and growth rates of future ovulatory follicles, diameters of the three largest follicles, and daily follicle count during the IOI are shown for each cycle and overall (Table 1). In this study, emergence of the future dominant follicle was detected during the IOI in 6 out of 9 spontaneous estrous cycles. For IOI 1 and overall, the growth rates from emergence to maximum diameter, Days –6 to –3, and Days –3 to –1 were all greater (*P* < 0.01) than the growth rate from maximum diameter to Day –1. Furthermore, the mean diameter of the largest follicle in IOI 2 was larger (*P* < 0.05) than in IOI 1. Regarding the daily follicle count, IOI 1 showed a greater (*P* < 0.05) number of small, 2–5 mm follicles compared to 20.1–25 mm follicles only. Moreover, IOI 2 had more (*P* < 0.01) 20.1–25 mm follicles than IOI 1. Overall, zebras had a relatively low number of antral follicles per IOI (3.1 ± 0.3, range: 2–4). Additionally, the mean diameter of the three largest follicles (Fig 3A), total number of follicles (Fig 3B), and number of follicles by diameter class (Fig 3C) from Day 0–32 after ovulation for all cycles combined (n = 9) are shown.

**Table 1:** Mean (± SEM) length of intervals, diameters, and growth rates of the future ovulatory follicle, diameters of the three largest follicles, daily follicle count for each class, and number of follicular waves during the interovulatory interval (IOI) in plains zebra (*Equus quagga*) mares.

End points	IOI 1 (Cycle 1)	IOI 2 (Cycle 2)	Overall
	(n = 4 cycles)	(n = 5 cycles)^§^	(n = 9 cycles)
Intervals (days) from:			
Ovulation to ovulation	38.5 ± 1.8	37.0 ± 1.7	37.7 ± 1.2
Ovulation to emergence at ≥ 6 mm	4.6 ± 1.2	6.5 ± 1.5	5.1 ± 1.0
Emergence to maximum diameter	32.6 ± 2.5	30.5 ± 0.5	32.0 ± 1.8
Emergence to ovulation	34.4 ± 2.5	32.0 ± 1.0	33.7 ± 1.8
Ovulation to maximum diameter	36.8 ± 1.8	35.4 ± 1.7	36.0 ± 1.2
Maximum diameter to ovulation	1.8 ± 0.3	1.6 ± 0.2	1.7 ± 0.2
Diameter of ovulatory follicle (mm):			
At emergence	6.8 ± 0.5	8.5 ± 0.5	7.3 ± 0.5
At maximum	37.3 ± 0.5	35.8 ± 0.6	36.4 ± 0.4
On Day –1	37.3 ± 0.5	35.8 ± 0.6	36.4 ± 0.4
Growth rates (mm/day) of the ovulatory follicle from:			
Emergence to maximum diameter	1.0 ± 0.1^a^	0.9 ± 0.0	1.0 ± 0.1^a^
Maximum diameter to Day –1	0.0 ± 0.0^b^	0.5 ± 0.5	0.1 ± 0.1^b^
Days –6 to –3	1.0 ± 0.1^a^	0.9 ± 0.1	0.9 ± 0.1^a^
Days –3 to –1	0.9 ± 0.2^a^	0.8 ± 0.1	0.8 ± 0.1^a^
Three largest follicles (mm)^†^			
F1 (largest)	19.7 ± 0.5^A^	22.7 ± 1.0^B^	21.6 ± 0.8
F2	7.6 ± 1.7	7.5 ± 1.6	7.5 ± 1.1
F3	5.8 ± 1.5	6.4 ± 1.6	6.2 ± 1.0
Daily follicle count during the IOI			
2–5 mm	1.4 ± 0.4^a^	0.6 ± 0.2	1.0 ± 0.3
5.1–10 mm	0.8 ± 0.3^ab^	0.5 ± 0.2	0.7 ± 0.2
10.1–15 mm	0.4 ± 0.2^ab^	0.3 ± 0.1	0.3 ± 0.1
15.1–20 mm	0.3 ± 0.1^ab^	0.4 ± 0.1	0.3 ± 0.1
20.1–25 mm	0.2 ± 0.0^A,b^	0.5 ± 0.2^B^	0.3 ± 0.1
>25 mm	0.4 ± 0.1^ab^	0.6 ± 0.1	0.5 ± 0.1
Total	3.4 ± 0.5	2.9 ± 0.3	3.1 ± 0.3
No. of follicular waves	0	0	0

A,BWithin a row, means with uncommon superscripts are different (*P* < 0.05). ^a,b^ Within a column, means with uncommon superscripts are different (*P* < 0.03–0.009). ^†^ Follicles were ranked each day without regard to day-to-day identity. ^§^ A third IOI was included for one zebra. Data were analyzed using either a one-way ANOVA followed by a Tukey test or a Kruskal-Wallis test followed by a Dunn’s test. Day 0 = ovulation; No. = number.

#### Ovulatory follicles.

**Fig 4:**
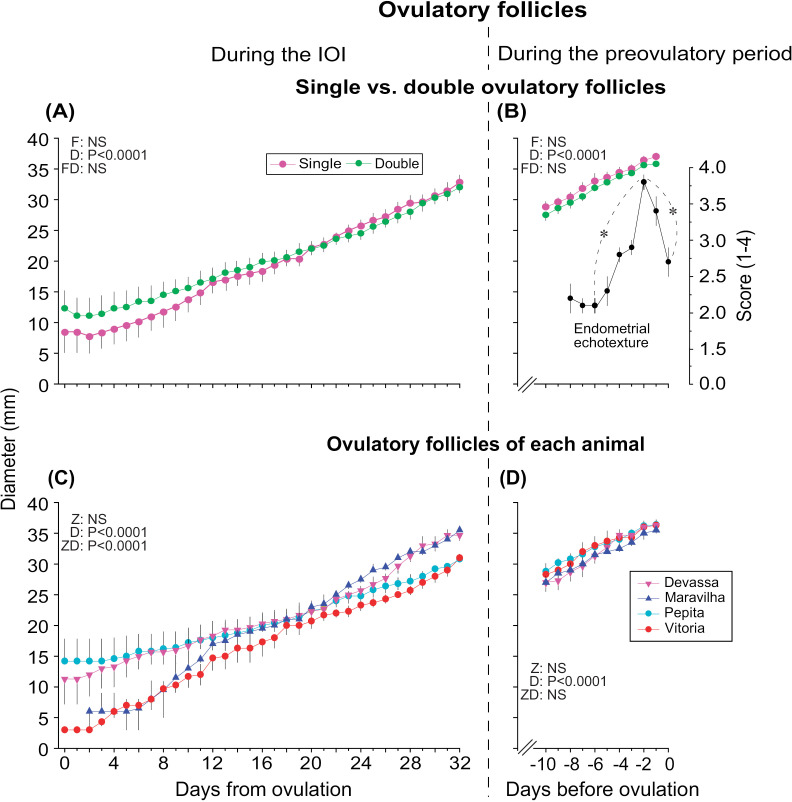
Diameters of ovulatory follicles and endometrial echotexture scores in plains zebra (*Equus quagga*) mares. Mean (± SEM) diameters of ovulatory follicles from (A, B) single vs. double ovulations and from (C, D) each animal (A, C) during the interovulatory interval (IOI) and (B, D) during the preovulatory period of spontaneous estrous cycles in plains zebra mares (n = 9 cycles in 4 zebras) are shown. Different colors indicate different (A, B) types of ovulations (single vs. double) and (C, D) zebra mares. (B) Mean (± SEM) endometrial echotexture scores for all spontaneous estrous cycles combined are also shown. The length of each IOI (indicated by the day of ovulation) varied between 33 and 41 days; therefore, the IOI for all cycles was truncated at Day 32 for statistical comparison. ^*^ Indicates the first significant increase and decrease (*P* < 0.05) when compared to the peak endometrial score. All data were transformed using rank or log_10_, then analyzed using one-way ANOVA followed by *post hoc* Tukey’s tests when applicable. F, follicle; D, day; FD, follicle by day interaction; NS, non-significant; Z, zebra; ZD, zebra by day interaction.

Mean diameters of ovulatory follicles for single (n = 5 cycles) and double (n = 4 cycles) ovulations are shown (Fig 4). The follicle diameters during the IOI (Days 0–32; Fig 4A and 4C) and preovulatory period (Days –10–0; Fig 4B and 4D) for zebras combined (Fig 4A and 4B) and individual zebras (Fig 4C and 4D) are pictured. Additionally, the mean endometrial echotexture score is also included during the preovulatory period for single and double ovulations combined (Fig 4B). The days of ovulation were Day 33 (n = 4 cycles), Day 38 (n = 2 cycles), Day 39 (n = 1 cycle), Day 40 (n = 1 cycle), and Day 41 (n = 1 cycle) for all zebras. During the IOI for single and double ovulations, the diameter of preovulatory follicles increased (*P* < 0.0001), with no other differences detected (Fig 4A). Moreover, during the preovulatory period, the diameter of the preovulatory follicle increased (*P* < 0.0001), regardless of single or double ovulation (Fig 4B). Additionally, the mean endometrial echotexture score during the preovulatory period was 2.7 ± 0.1, with a significant increase (*P* < 0.0001) in score between Day –5 (2.1 ± 0.1) and Day –2 (3.8 ± 0.1) and a decrease (*P* < 0.0001) from Day –2 until Day 0 (2.7 ± 0.2). Regarding the diameter of preovulatory follicles per zebra during the IOI (Fig 4C), the diameter of the preovulatory follicles increased (*P* < 0.0001), but was not different (*P* > 0.05), between zebras. Nonetheless, interaction was detected (*P* < 0.0001) between zebras and days, indicating that follicles from the zebras grew at different rates during the IOI. When considering the mean diameter of preovulatory follicles during the preovulatory period (Fig 4D), the diameter of follicles increased (*P* < 0.0001) until the day before ovulation, regardless of zebra. However, unlike during the IOI, no interaction (*P* > 0.05) was detected during the preovulatory period. On the day of spontaneous ovulation (Day 0), the mean CL diameter was 30.7 ± 0.4 mm (range, 29–33 mm).

#### Individual tracked follicles per zebra.

**Fig 5:**
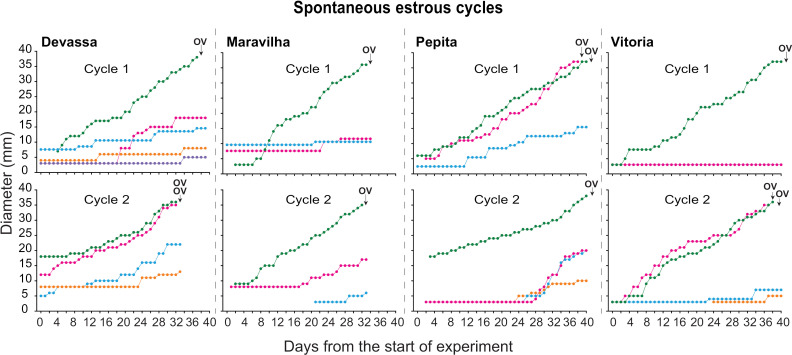
Diameters of all antral follicles during two spontaneous estrous cycles in four plains zebra (*Equus quagga*) mares. All follicles were tracked from the start of ultrasonographic scanning until ovulation of the preovulatory follicle(s). Different colors indicate different individual follicles. Arrow indicates day of ovulation, with synchronous double ovulations superimposed (i.e., Devassa, Cycle 2). OV, ovulation day.

The diameter profile during the IOI of all tracked follicles for two consecutive spontaneous estrous cycles in four zebras is shown (Fig 5). One zebra (Pepita) had a third estrous cycle (S1 Fig), in which a double ovulation was displayed, with a static follicular growth rate 2 days prior to ovulation. Interestingly, in the 3 cycles where emergence was not detected during the IOI, it appeared as though the future dominant follicle seemed to emerge during middle to end of the previous spontaneous estrous cycle. Additionally, for all the spontaneous cycles tracked, 66.7% (6 out of 9) showed a static preovulatory follicle growth phase 2–3 days prior to ovulation. Furthermore, 4 out of 9 cycles displayed co-dominance and double ovulation, with only one zebra mare (Maravilha) not experiencing a double ovulation in any cycle.

### Experiment 2. Induced estrous cycles

#### Follicle dynamics.

The mean intervals (in days) of key stages of ovulation induction (PG and hCG administration, maximum diameter, ovulation, etc.), diameters and growth rates of future ovulatory follicles, diameters of the three largest follicles, and daily follicle count for two zebras (Vitoria and Pepita) and overall, during the induction period (n = 10 cycles), are shown (Table 2). Regarding intervals, the interval for F1 at maximum diameter to ovulation tended to be longer (*P* = 0.07) for the zebra Vitoria than for Pepita. Furthermore, Vitoria had a larger (*P* < 0.001) ovulatory follicle diameter than Pepita for all time points evaluated (at PG, at hCG, at maximum, and on Day –1). Moreover, while the ovulatory follicles of Pepita grew at a steady, similar (*P* > 0.05) rate throughout the induced ovulation time period, Vitoria’s ovulatory follicle growth rate differed depending on the time point. The growth rate of Vitoria’s ovulatory follicle from administration of PG until administration of hCG was greater (*P* < 0.05) than the growth rate after hCG was administered until Day –1. Additionally, the growth rate of Vitoria’s ovulatory follicle from Days –6 to –3 was greater (*P* < 0.05) than the growth rate from Days –3 to –1 and from administration of hCG until Day –1. This is mirrored in the overall cycles, as the ovulatory follicle growth rate from administration of PG until administration of hCG was greater (*P* < 0.05) than the growth rate after hCG was administered until Day –1. Furthermore, the growth rate from Days –6 to –3 was greater (*P* < 0.05) than the growth rate from Days –3 to –1 and from administration of hCG until Day –1. Moreover, in both zebras, preovulatory follicles in induced cycles did not grow from maximum diameter until Day –1.

**Table 2:** Mean (± SEM) length of intervals, diameters, and growth rates of the future ovulatory follicle, diameters of the three largest follicles, and daily follicle count for each class from prostaglandin administration to Day –1 in plains zebra (*Equus quagga*) mares.

End points	Zebra “Vitoria”	Zebra “Pepita”	Overall
	(n = 5 cycles)	(n = 5 cycles)	(n = 10 cycles)
Intervals (days) from:			
Start of experiment to PG	7.2 ± 1.0	7.4 ± 0.2	7.3 ± 0.5
PG to hCG	3.6 ± 0.5	3.0 ± 0.0	3.3 ± 0.3
PG to ovulation	5.6 ± 0.5	4.8 ± 0.2	5.2 ± 0.3
hCG to ovulation	2.2 ± 0.2	1.8 ± 0.2	2.0 ± 0.1
PG to F1 at maximum diameter	4.4 ± 0.4	3.6 ± 0.2	4.0 ± 0.3
F1 at maximum diameter to ovulation	1.8 ± 0.2^#^	1.2 ± 0.2	1.5 ± 0.2
Diameter of ovulatory follicle (mm):			
At PG	25.2 ± 0.5^A^	20.6 ± 0.6^B^	22.9 ± 0.8
At hCG	36.4 ± 0.2^A^	25.8 ± 0.4^B^	31.1 ± 1.8
At maximum	36.8 ± 0.2^A^	26.4 ± 0.2^B^	31.6 ± 1.7
On Day –1	36.8 ± 0.2^A^	26.4 ± 0.2^B^	31.6 ± 1.7
Growth rates (mm/day) of the ovulatory follicle from:			
Start of experiment to PG	2.0 ± 0.3^abc^	1.3 ± 0.2	1.6 ± 0.2^a^
PG to hCG	2.3 ± 0.4^ab^	1.5 ± 0.2	2.0 ± 0.2^ab^
PG to Day –1	2.2 ± 0.4^abc^	1.5 ± 0.2	1.9 ± 0.2^ab^
hCG to Day –1	0.3 ± 0.2^c^	0.8 ± 0.3	0.5 ± 0.2^c^
Maximum diameter to Day –1	0.0 ± 0.0	0.0 ± 0.0	0.0 ± 0.0
Days –6 to –3	2.9 ± 0.3^A,b^	1.7 ± 0.3^B^	2.3 ± 0.2^b^
Days –3 to –1	1.1 ± 0.3^ac^	1.1 ± 0.2	1.1 ± 0.2^ac^
Three largest follicles (mm)^†^			
F1 (largest)	24.5 ± 0.5^A^	18.7 ± 0.6^B^	21.6 ± 0.7
F2	12.4 ± 0.6	11.6 ± 0.9	11.8 ± 0.3
F3	11.9 ± 1.0	9.7 ± 1.1	10.8 ± 0.3
Daily follicle count from PG to Day –1			
2–5 mm	0.0 ± 0.0	0.4 ± 0.4^b^	0.2 ± 0.2^b^
5.1–10 mm	1.3 ± 0.4^b^	1.0 ± 0.3^a^	1.2 ± 0.2^c^
10.1–15 mm	0.8 ± 0.2^ab^	1.0 ± 0.1^a^	0.9 ± 0.1^ac^
15.1–20 mm	0.4 ± 0.1^ab^	0.4 ± 0.1^ab^	0.4 ± 0.1^ad^
20.1–25 mm	0.2 ± 0.0^a^	0.2 ± 0.0^b^	0.2 ± 0.0^bd^
>25 mm	0.4 ± 0.0^A,ab^	0.1 ± 0.0^B,b^	0.3 ± 0.1^bd^
Total	3.1 ± 0.2	3.2 ± 0.3	3.2 ± 0.2

A,BWithin a row, means with uncommon superscripts are different (*P* < 0.05). ^a,b^ Within a column, means with uncommon superscripts are different (*P* < 0.02–0.0001). ^#^ Within a row, the mean values of each zebra tended to be different (*P* = 0.07). ^†^ Follicles were ranked each day without regard to day-to-day identity. Data were analyzed using either a one-way ANOVA followed by a Tukey test or a Kruskal-Wallis test followed by a Dunn’s test. PG, prostaglandin. hCG, human chorionic gonadotropin. Day 0 = ovulation. F1, largest follicle.

When comparing the ovulatory follicles between zebras, Vitoria’s ovulatory follicle growth rate from Days –6 to –3 was greater (*P* < 0.05) than Pepita’s. Furthermore, Vitoria had a greater (*P* < 0.03) growth rate during the preovulatory follicle period (Days –6 to –1; growth rate = 2.4 ± 0.2) than Pepita (growth rate = 1.5 ± 0.2). Additionally, the diameter of Vitoria’s largest follicle (F1) was greater (*P* < 0.05) than that of Pepita’s largest follicle during the ovulation induction period. Finally, regarding the daily follicle count from PG administration until Day –1, different zebras showed different patterns.

#### Individual tracked follicles per zebra.

**Fig 6:**
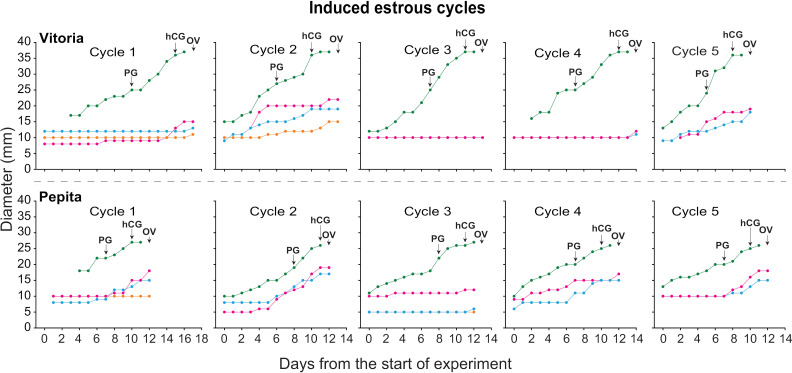
Diameters of all antral follicles during five induced ovulatory cycles in two plains zebra (*Equus quagga*) mares (n = 10 cycles total). All follicles were tracked from the start of ultrasonographic scanning until ovulation of the preovulatory follicle. Different colors indicate different individual follicles. Arrows indicate days of exogenous drug administration or ovulation. PG, prostaglandin analogue (dinoprost tromethamine; Lutalyse) administration; hCG, human chorionic gonadotropin (Vetecor) administration; OV, ovulation day.

The diameter profile of all tracked follicles in two zebras for 5 induced cycles (n = 10 cycles total) each is shown (Fig 6). Of the induced cycles tracked, 50% showed a static phase 2 days prior to ovulation, with no cycle showing co-dominance and double ovulation, unlike the spontaneous cycles. On the day of ovulation (Day 0) for the induced cycles, the mean CL diameter was 27.4 ± 1.4 mm (range, 22–34 mm).

### Comparison of spontaneous and induced ovulatory cycles

**Fig 7:**
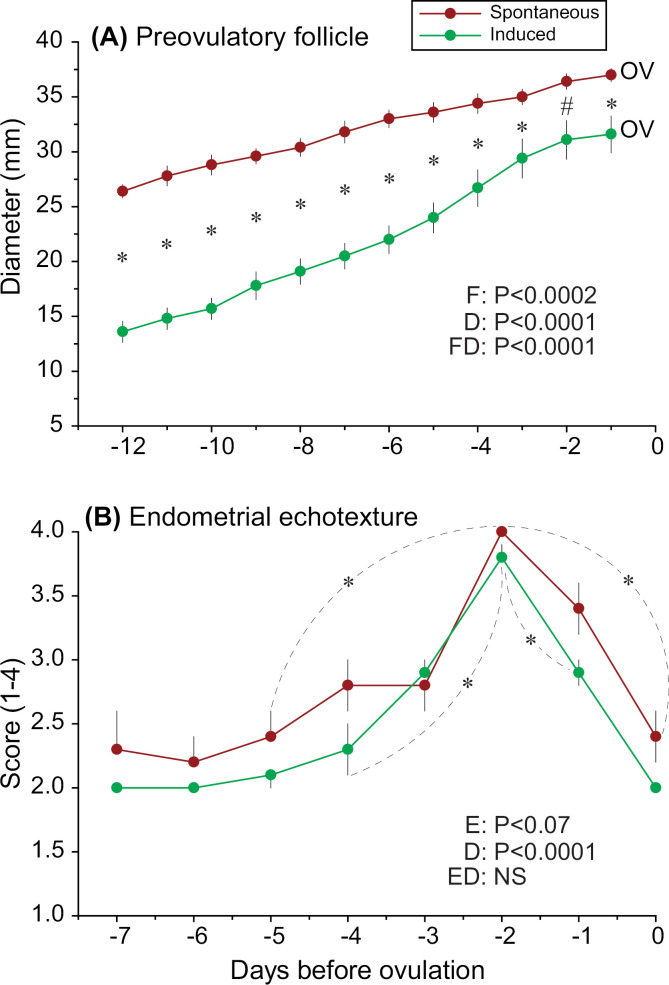
Diameters of preovulatory follicles and endometrial echotexture scores during spontaneous estrous and induced ovulatory cycles in single-ovulating plains zebra (*Equus quagga*) mares. Mean (± SEM) (A) preovulatory follicle diameter and (B) endometrial echotexture score for spontaneous estrous and induced ovulatory cycles in single-ovulating plains zebra mares (n = 5 spontaneous cycles in 4 zebras and n = 10 induced cycles in 2 zebras) are shown. Different colors indicate different types of cycle (spontaneous vs. induced). ^*^ Indicates significant differences (*P* < 0.05) in (A) follicular diameter between type of cycle within the same day, and (B) the first significant increase and decrease when compared to the peak endometrial echotexture score within the same type of cycle. ^#^ Indicates a tendency (*P* < 0.1) for a difference in follicular diameter. All data were transformed using rank or log_10_, then analyzed using one-way ANOVA followed by *post hoc* Tukey’s tests when applicable. OV, day of ovulation; F, follicle; D, day; FD, follicle by day interaction; E, endometrial score; ED, endometrial score by day interaction; NS, non-significant.

The growth of preovulatory follicles and endometrial echotexture score during the preovulatory period of spontaneous and induced ovulatory cycles in single-ovulating zebras is shown (Fig 7). The diameter of spontaneous preovulatory follicles (n = 5 cycles, n = 5 follicles) was larger (*P* < 0.0002) than that of induced follicles (n = 10 cycles, n = 10 follicles) during the preovulatory period (Fig 7A). Additionally, for both groups, follicular diameter increased (*P* < 0.0001) during the overall preovulatory period. Furthermore, interaction was detected (*P* < 0.0001) between group and day, due to an increased growth rate of induced ovulatory follicles when compared to spontaneous. In this regard, induced ovulatory follicles showed a greater (*P* < 0.0001) growth rate (1.9 ± 0.2) than spontaneously ovulating follicles (0.8 ± 0.1) between Days –6 to –1. Moreover, from Days –6 to –3, the growth rate of follicles from induced ovulations (2.3 ± 0.3) was greater (*P* < 0.0001) than in spontaneously ovulating follicles (0.9 ± 0.1). However, the growth rate of ovulatory follicles from maximum diameter until Day –1 was similar (*P* > 0.05) between spontaneous and induced ovulatory cycles.

The overall endometrial echotexture score during the preovulatory period of zebras with spontaneous ovulations (2.8 ± 0.1) in single-ovulating cycles tended to be higher (*P* < 0.07) than those from induced ovulatory cycles (2.6 ± 0.1; Fig 7B). Both spontaneous and induced zebras showed a peak in endometrial echotexture 2 days prior to ovulation. For zebras with spontaneous ovulations, there was a significant increase (*P* < 0.0001) in endometrial echotexture between Days –5 (2.4 ± 0.2) and –2 (4.0 ± 0.0). Meanwhile, in induced ovulations, this increase (*P* < 0.0001) occurred between Days –4 (2.3 ± 0.2) and –2 (3.8 ± 0.1). The endometrial echotexture peak was then followed by a decrease (*P* < 0.0001) in score for both groups, with lower (*P* < 0.0001) endometrial echotexture being observed by Day –1 (2.9 ± 0.1) in induced ovulations, and Day 0 (day of ovulation; 2.4 ± 0.2) in spontaneous.

## Discussion

This is the first study in any zebra species to utilize transrectal ultrasonography to track and describe the *in vivo* dynamics of follicular growth, ovulation, and changes in endometrial echotexture over time. Data collected via tracking two spontaneous estrous cycles in four cycling zebra mares revealed that the estrous cycle of the plains zebra mare has distinct differences from and intriguing similarities to the estrous cycles of domestic equids like the horse mare and jenny. Overall, the spontaneous estrous cycles of the plains zebra mare were characterized by a long IOI, a low daily antral follicle count during the IOI, an absence of follicular waves, and similar preovulatory follicle diameters to the jenny. Furthermore, the plains zebra mare shows endometrial echotexture patterns similar to those of the horse mare during the preovulatory period. Additionally, this is the first study to evaluate the use of ovulation induction drugs (PG and hCG) in the zebra. Through the evaluation of five induced ovulatory cycles in two plains zebra mares, it was proven that these animals do respond to PG and hCG and can therefore be short-cycled and induced to ovulate in a very controlled, manageable way. Altogether, these findings show key similarities and differences in estrous cycle patterns between the plains zebra and other equids that must be considered when designing breeding programs and developing ARTs in this vital species.

### Comparison of IOIs

The plains zebra mare shows a much longer IOI (37.7 days) than the horse mare (20.3–23.9 days; [12,35,36]), jenny (23.0–27.3 days, depending on season; [11,37,38]), and even other endangered species like the Persian onager (22–28 days, [39]) and the Przewalski’s horse (21–24 days; [40]). However, similar to horse mares and jennies, there was a wide range of IOIs between individual zebras and cycles (33–41 days in the zebra mare; 19–26 days in the horse mare, [41,42]; 21–28 days in the jenny, [38,43]). Interestingly, the day interval of the previous ovulation to emergence in the plains zebra mare was about 5 days, very similar to previous findings in Criollo mares [15] and jennies [11]. Furthermore, the plains zebra mare ovulated around 1–2 days after the maximum diameter of the preovulatory follicle was reached, matching what has been reported in the horse mare [15,19]. Therefore, the increased IOI seen in the plains zebra mare is likely due a slower growth rate (approximately 1 mm per day) from emergence until the dominant/ovulatory follicle reaches maximum diameter, whereas the growth rate for this same aspect in horse mares and jennies is approximately 2–3 mm per day [11,12,15,38]. Nonetheless, despite the plains zebra having a slower follicular growth rate from emergence until maximum diameter, a static or reduced growth phase of the preovulatory follicle until ovulation was observed in a majority of the tracked spontaneous cycles (6 out of 9 cycles), which is a common phenomenon similarly found in horse mares [12,15] and jennies [11,44]. Therefore, even though the plains zebra mare has a longer IOI due to a slower ovulatory follicle growth rate, we hypothesize that the mechanisms of follicular emergence, growth, and ovulation are very similar between the plains zebra, the horse, and the donkey. However, this longer IOI and slow follicular growth rate in the zebra mare can be ameliorated and controlled using exogenous drugs like PG and hCG to induce luteolysis and ovulation, respectively.

### Use of exogenous drugs to control IOI

Overall, through the use of PG and hCG following industry standards for horse mares [32,45] and jennies [46], luteolysis and ovulation in the zebras included in this study were able to be successfully induced in all attempts (n = 10 cycles from two plains zebra mares). While some variation regarding day interval was seen within and between zebras, luteolysis and ovulation can generally be controlled. Overall, the effects of luteolysis (i.e., increased endometrial edema) began to occur around 3 days after PG administration (5–6 days before ovulation), and ovulation occurred around 2 days after hCG administration. While PG-induced luteolysis, indicated by decreased plasma progesterone levels (< 2 ng/ml in serum), begins relatively rapidly in the mare (24–48 h; [47,48]), it can take at least 72 hours to see such progesterone decreases in jennies [49]. It should be noted that neither plasma progesterone levels at any point nor CL diameter after the day of ovulation were analyzed in the current study; however, endometrial edema score via ultrasonographic echotexture evaluation was included as a proxy for this analysis. While the mechanisms of PG-induced luteolysis in the plains zebra mare are unknown and, at the moment, difficult to compare to the horse mare and jenny, all three of these equid species appear to ovulate within 24–48 h after proper hCG administration [32,50]. This may suggest a similar mechanism of action of hCG-induced ovulation between equid species, as this study demonstrates that administering hCG approximately 3 days after PG can successfully induce ovulation in the plains zebra mare in around 2 days. Therefore, the use of PG and hCG in plains zebra mares can both reduce the time between consecutive ovulations and reliably induce ovulations, which is essential for developing ARTs in species with long, spontaneous IOIs.

### Comparison of follicular characteristics

Despite the much longer IOI of the zebra mares observed in this study, the plains zebra mare ovulates a slightly smaller follicle (mean, 36.4 mm) than horse mares (means range, 38.3–44.5 mm, depending on breed; [12,15], but one that is of a comparable size to that of jennies (means range, 34.6–36.9 mm, depending on breed; [11,16,38]). While dominant follicle diameters have been reported in other wild equid species, such as the Przewalski’s horse [40] and the Persian onager [39], these data were collected every 3 days and, therefore, do not provide a precise measurement of the follicular diameter one day before ovulation. Interestingly, even though the growth rate of the ovulatory follicle in induced cycles increased to 2 mm per day until Day –3, the diameter of the ovulatory follicle in induced cycles was smaller than in spontaneous cycles at all times. This smaller induced ovulatory follicle diameter is similarly seen in horse mares [51] and has been suggested to occur in jennies [31]. Moreover, unlike most domestic equids, the plains zebra mare does not appear to show the traditional follicular waves that are classically observed in the horse mare [12] and jenny [10,11]. While clear patterns of emergence, selection, and dominance in primary and secondary waves can be seen in the horse mare and jenny [10–12], no synchronic growth and regression of follicles with clear selection of a dominant follicle could be seen in spontaneous or induced cycles using daily transrectal ultrasonography in the plains zebra mare. This is likely due, in part, to the low daily follicle count per cycle (i.e., 3 follicles), a much lower number than seen in larger horse mare breeds (10–15 follicles; [12,15] and jennies (12.5 follicles; [11]). Interestingly, the miniature pony breed of horse mare also has a low daily follicle count per cycle, around 1.5 follicles, and, while follicular waves were present and classified in 55% of cycles, the follicular waves observed followed less classical and predictable patterns than in larger breeds of horse mares [19]. Therefore, while the current study did not find any pattern indicating the presence of classical follicular waves seen in larger horse mare breeds and jennies, future follow-up studies using a larger number of zebra mares across consecutive IOIs and including hormonal profiles (i.e., FSH, LH) should be performed to confirm this finding.

Finally, 4 double ovulations out of 9 cycles (44%) were observed in spontaneous plains zebra estrous cycles. The initial finding of such a high percentage of double ovulations in spontaneous cycles was surprising, as the incidence of twin births in any zebra species is incredibly rare [1,3,52]. Therefore, a similar or even higher number of double ovulations was expected in induced cycles, as the use of PG to induce luteolysis has been linked to an increased frequency of double ovulations in horse mares [53]. However, no double dominance/preovulatory follicles or double ovulations were observed in any induced ovulatory cycles in the present study. Follow-up studies are therefore essential to determine if the high incidence of double ovulations in spontaneous plains zebra estrous cycles and lack of double ovulations in induced cycles is a species-wide trait or if this was particular to our sample group.

### Endometrial echotexture

Regardless of spontaneous estrous or induced ovulatory cycles, the endometrial echotexture during the preovulatory period of the plains zebra showed a similar pattern of increasing significantly 4–5 days before ovulation, then decreasing significantly 1–2 days before ovulation. This is similar to what is observed in the horse mare [15,19,51,54] and, to a degree, can be used to better predict ovulation and facilitate breeding in captivity. However, the jenny does not appear to exhibit a reliable decrease in endometrial echotexture pattern prior to ovulation [11,31,55]. This highlights that, although the plains zebra mare shares several estrous cycle characteristics with the jenny, some characteristics are more similar to those of the horse mare and emphasizes the importance of targeted study on the reproduction of this key species.

### Applications, limitations, and next steps

Understanding follicular dynamics during the estrous cycle is essential for the development of breeding programs and ARTs in any species. In conservation settings, where populations are small or shrinking and breeding logistics are complex (e.g., genetic and reproductive compatibility, animal transportation; [22]), this knowledge becomes even more time-sensitive for rescuing these critical species. Consequently, studies that evaluate reproductive physiology in species like the plains zebra are particularly appealing, as these can provide comparative information regarding this species and other, more threatened zebra species like the mountain and Grévy’s zebras [6–8]. However, because handling these wild equids is extremely difficult, the development of ARTs has lagged behind that of their domestic counterparts [25]. Therefore, findings of this study, such as the IOI length, different aspects related to follicular dynamics, and endometrial echotexture changes before ovulation, provide key information that can be used to improve reproductive efficiency and preservation of the plains zebra. These can not only help in predicting when to best expose a plains zebra mare to a zebra stallion, but they can also assist in the development of AI protocols. The development of AI programs is particularly appealing for eliminating the transport of breeding animals, relying solely on semen, and enabling the potential expansion of the breeding gene pool beyond the limits of animal transport [22]. Furthermore, this information, paired with findings that the plains zebra estrous cycle can be manipulated with PG and hCG, has tremendous potential for developing timed AI protocols and optimizing OPU and embryo transfer protocols. Additionally, these findings serve as a putative comparative model for other zebra species. However, it must be noted that some inter-zebra species differences are expected, given the variation in follicular development seen in breeds of horse mares (Quarter Horses, Clydesdales, Belgians, and Arabians, [33]; miniature ponies, [19]; larger ponies and Breton crosses, [12]; Criollo, [15]) and jennies (Brazilian Northeastern, [31]; Caribbean, [11]; Dezhou black, [10]; Egyptian, [44]; Mexican Burro, [38]; Catalonian, Poitou, Pega, Mammoth, and North African, [16]).

Future studies that explore follicular dynamics and ovulation during the estrous cycles of other zebra species are warranted to explore these presumed comparative aspects. Unfortunately, data on CL characteristics and plasma hormone concentrations could not be collected in the present study due to behavioral and time constraints of the plain zebra mares. Therefore, studies that evaluate these key aspects of the zebra estrous cycle are essential for further characterizing ovarian cyclicity in this species. It is of note that, through extensive training, all zebra mares evaluated were able to be scanned daily via transrectal ultrasonography in standard equid palpation chutes without sedation. Therefore, in future studies, larger groups of plains zebra mares could be potentially trained to undergo frequent blood sampling and more detailed daily ultrasonography ovarian data collection to provide additional information on the reproductive physiology of this species.

## Conclusions

The present study reports, for the first time, the follicular dynamics and endometrial echotexture changes of spontaneous estrous and induced ovulatory cycles in plains zebra mares. Furthermore, this is the first study to evaluate the use of exogenous drugs to control the zebra estrous cycle via induction of luteolysis and ovulation. The following characteristics were observed: (i) a long IOI (approx. 38 days) paired with a slow growth rate (approx. 1 mm per day) and relatively similar ovulatory follicle diameter (approx. 36 mm; range, 31–41 mm) compared to domestic equids in spontaneous estrous cycles and (ii) that the plains zebra can respond to luteolytic and ovulation synchronization drugs in a reliable way that can be used to shorten the overall cycle length and induce ovulation. Furthermore, it was revealed that the endometrial echotexture of the plains zebra mare during the preovulatory period changes in patterns similar to those of the horse mare, which is a trait that does not seem to be shared with the jenny. Overall, the information provided in this study is vital for the development of breeding programs and ARTs in the plains zebra and can be potentially applied to other, more endangered zebra species.

## Supporting information

S1 Fig Diameters of all antral follicles during the third tracked spontaneous estrous cycle in a plains zebra (*Equus quagga*) mare, “Pepita. **”** All follicles were tracked from the start of ultrasonographic scanning until ovulation of the preovulatory follicles. Different colors indicate different individual follicles. The arrow indicates days of ovulation. OV, ovulation day.
(EPS)

S1 File (XLSX)
